# Performance of mixed effects models in the analysis of mediated longitudinal data

**DOI:** 10.1186/1471-2288-10-16

**Published:** 2010-02-19

**Authors:** Emily A Blood, Howard Cabral, Timothy Heeren, Debbie M Cheng

**Affiliations:** 1 Department of Biostatistics, Boston University School of Public Health, 715 Albany Street Crosstown Center-3rd Floor Boston, MA 02118, USA; 2 Clinical Research Program, Children's Hospital Boston and Harvard Medical School, 300 Longwood Avenue Boston, MA 02115, USA

## Abstract

**Background:**

Linear mixed effects models (LMMs) are a common approach for analyzing longitudinal data in a variety of settings. Although LMMs may be applied to complex data structures, such as settings where mediators are present, it is unclear whether they perform well relative to methods for mediational analyses such as structural equation models (SEMs), which have obvious appeal in such settings. For some researchers, SEMs may be more difficult than LMMs to implement, e.g. due to lack of training in the methodology or the need for specialized SEM software. It therefore is of interest to evaluate whether the LMM performs sufficiently in a scenario particularly suitable for SEMs. We focus on evaluation of the total effect (i.e. direct and indirect) of an exposure on an outcome of interest when a mediating factor is present. Our aim is to explore whether the LMM performs as well as the SEM in a setting that is conducive to using the SEM.

**Methods:**

We simulated mediated longitudinal data from an SEM where a binary, main independent variable has both direct and indirect effects on a continuous outcome. We conducted analyses with both the LMM and SEM to evaluate the performance of the LMM in a setting where the SEM is expected to be preferable. Models were evaluated with respect to bias, coverage probability and power. Sample size, effect size and error distribution of the simulated data were varied.

**Results:**

Both models performed well in a range of settings. Marginal increases in power estimates were observed for the SEM, although generally there were no major differences in performance. Power for both models was good with a sample of size of 250 and a small to medium effect size. Bias did not substantially increase for either model when data were generated from distributions that were both skewed and kurtotic.

**Conclusions:**

In settings where the goal is to evaluate the overall effects, the LMM excluding mediating variables appears to have good performance with respect to power, bias and coverage probability relative to the SEM. The major benefit of SEMs is that it simultaneously and efficiently models both the direct and indirect effects of the mediation process.

## Background

A common method of handling longitudinal data is through linear mixed effects models (LMMs) [1]. These models account for the correlation of observations and allow estimation of the effect of predictor variables on repeated outcomes. They are relatively easy to implement and their regression parameters have a clear interpretability.

Complex relationships often exist among the variables studied, however, and it may be of interest to explicitly model the hypothesized causal pathways between independent variables and outcomes. Although multiple mixed effects models can be fit to evaluate mediation (see e.g. Krull and MacKinnon [2] and Baron and Kenny [3]), methods for mediational analyses, such as Structural Equation Models (SEMs), are necessary to simultaneously model mediated relationships. However, when the primary aim of an analysis is to determine the total effect (i.e. direct and indirect) of an exposure on an outcome of interest, it is unclear what the impact of explicit modeling of the mediated relationship is on power, bias, and on coverage probability for the main research aim.

SEMs are a well known and commonly used data analysis technique in the social sciences, and is becoming increasingly popular in many clinical research areas. The SEM framework is a general modeling framework and allows the modeling of potentially complex relationships among observed and latent variables and can be applied in the longitudinal data setting.

There has been much previous work in applying SEMs to longitudinal data analyses [4-9], and the equivalence of LMMs and special cases of SEMs in settings without mediating variables has been well documented in the SEM literature [5,7,10-15]. The major advantages of SEMs are that they have the capability to incorporate measurement error on the variables in the model [5,8,13], allow explicit modeling of relationships involving mediating variables [16] and are able to decompose direct and indirect effects [11]. Disadvantages of SEMs that have been previously noted include potentially large sample size requirements and potential problems with skewed data. It is also essential for SEMs that investigators have clear hypotheses on the causal pathways between variables [9]. In addition, SEMs may be more difficult than LMM to implement, e.g. due to a lack of training in the methodology or the need for specialized SEM software. Given these potential limitations, it is of interest to explore whether a LMM performs well relative to an SEM in settings where mediation is present.

The purpose of this paper is to conduct a simulation study in a mediated longitudinal setting to evaluate whether a LMM performs sufficiently with respect to power, bias and coverage in a scenario that is conducive to using an SEM.

## Methods

### Setting

**Figure 1:**
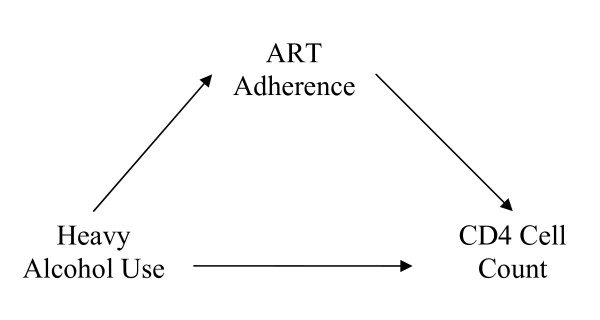
**Mediated effect of alcohol on CD4 count**. Alcohol may directly impact CD4 count or may have an indirect effect through its effects on ART adherence.

We consider a longitudinal setting similar to a study by Samet et al [17] evaluating the impact of heavy alcohol consumption on HIV disease progression. The data arise from a prospective cohort study in which the primary outcome, CD4 cell count, is assessed every 6 months for three years (i.e. 6 measures of CD4 count across time for each subject) and heavy alcohol consumption, the main independent variable, is assessed only at baseline. A potential mediator of the relationship between heavy alcohol consumption and HIV disease progression is adherence to antiretroviral therapy (ART) as it has been demonstrated that alcohol consumption may worsen a patient's ability to adhere to ART thereby leading to worse disease progression. In the current setting we assume that ART is assessed only at baseline. In addition to an indirect effect mediated by ART, alcohol consumption could also have a direct biological effect on CD4 cell count. The primary objective of the analysis is to evaluate the overall impact (direct plus indirect effect) of heavy alcohol use on CD4 cell count. Figure 1 shows a simple diagram illustrating the relationship between heavy alcohol consumption, ART adherence and the outcome CD4 cell count.

A standard analytic approach for analyzing these data would be to fit a LMM, which can account for correlation due to repeated assessments of CD4 cell count from the same subject and adjust for potential confounders. Alternatively, an SEM could be fit to the data which would explicitly model the hypothesized pathways between heavy alcohol consumption and CD4 count.

In this setting, where the main objective is to determine the total effect of heavy alcohol use on CD4 cell count, it is unclear whether a LMM can perform as well as an SEM, a method often preferred for analyzing mediated longitudinal data.

### General SEM Formulation

There are two components to an SEM, the measurement model and the structural model. The measurement model relates unobserved latent variables and covariates to outcomes and exposure indicators. This model attempts to capture measurement error in observed variables. The structural model relates covariates and latent variables to latent variables. This model attempts to capture individual variation in the latent variables.

Using the same notation as Sanchez [15], the general model is expressed as:

Measurement Model(1)

Structural Model(2)

In the above equations, *i *indexes the individual, with *i *= 1,..., *N *where *N *is the number of individuals. For the *i*th individual, **Y_i _**is a vector of observed outcomes, **X_i _**is a vector observed exposure indicators. **U_i _**is a vector of latent variables and **Z_i _**is a vector of observed, fixed covariates. Although **U_i _**appears on both sides of the matrix equation, the diagonal elements of **B **are zeros so that the same element of **U_i _**would not appear on both the left- and right-hand side of a given equation. is a matrix of coefficients associated with **U_i_**, **K **is a matrix of coefficients associated with **Z_i _**and *ε*_i _is a vector of random residual errors for the measurement model. In the structural model, **B **is a matrix of coefficients (where the diagonal elements are zeros) associated with **U_i_**, Γ is a matrix of coefficients associated with **Z_i _**and *ζ*_*i *_is a vector of random residual errors for the structural part of the model. The mean of random residual errors for both the measurement and structural models are assumed to be zero. **Σ **is the covariance matrix of the residual errors of the measurement model (*ε*_*i*_), and **Ψ **is the covariance matrix of the residual errors of the structural model (*ζ*_*i*_). The **X_i _**and **Y_i _**are assumed to be multivariate normal (MVN). The errors in Equations 1 and 2 are assumed to be independent. Parameters are usually estimated via maximum likelihood, with the objective of minimizing the distance between the observed and model-based mean and covariance structure [16].

### SEM Simulation Model

**Figure 2:**
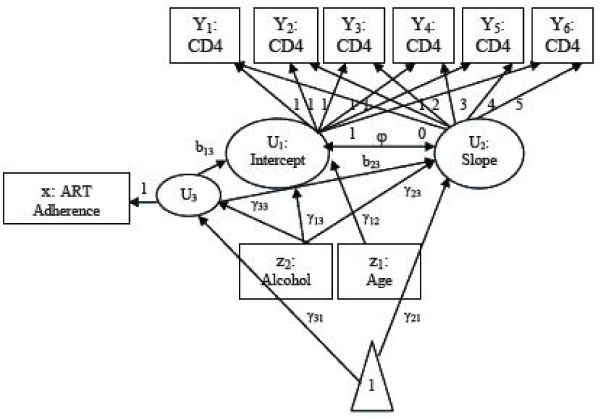
**Path diagram for mediated longitudinal data**. path diagram for mediated longitudinal data with the outcome (*Y*) measured at six occasions, a continuous covariate (*z*_1_) measured once, a dichotomous main independent predictor (*z*_2_) measured once variable and a continuous mediator (*x*) measured once.

The SEM framework was used to generate the mediated longitudinal data for the simulation studies since the aim is to evaluate whether a LMM performs sufficiently in the setting where an SEM is presumed to be optimal. The scenario in which we simulated data is an extension of a specific SEM often referred to as a latent growth curve model or latent curve model [4,18]. In the latent growth curve model, the outcome variables are influenced by random intercept and slope variables. These variables are latent and can be influenced by predictors and other covariates. Let *i *index the individual (*i *= 1,..., *N*) and *j *index the time-point (*j *= 1,..., *T*), where *T *is the number of measurement times. In the current study, we considered a setting with a single continuous covariate (*z*_1*i*_), such as age, and a single binary independent variable (*z*_2*i*_) of primary interest, heavy alcohol consumption, predicting repeated observations of the outcome (*Y*_*ij*_), CD4 cell count. Heavy alcohol use influences the outcome CD4 cell count through the random intercept and slope variables. In addition, ART adherence is a mediating variable (*x*_*i*_) which influences CD4 count through the random intercept and slope variables. The variable ART adherence is said to be a mediator because the primary independent variable, heavy alcohol use, may affect CD4 count not only directly but also indirectly through ART adherence. We considered a setting with 6 time-points (*T *= 6) and illustrate the SEM model with the path diagram in Figure 2. Using the notation we have described above for SEMs and eliminating the subject index *i *for simplicity, our measurement and structural model for the scenario illustrated in Figure 2 can be written as:

Measurement Model(3)

Structural Model(4)

or in matrix notation as:

Measurement Model

Structural Model

The latent intercept and slope are represented by *U*_1 _and *U*_2_, respectively. The continuous covariate (age) and main independent variable (heavy alcohol use) are represented by *z*_1 _and *z*_2_, respectively. Based on the model formulation presented in Equations 1 and 2, ART adherence (*x*) is considered both an outcome (as it is influenced by the main independent variable) and a predictor (as it influences the random intercept and slope), and therefore appears on the right- and left-hand side of the above equations. However, using the above formulation, only latent variables can be both outcomes and predictors. Thus to incorporate *x *as a mediator and stay within the framework defined by Sanchez [15], we must add an additional latent variable (*U*_3_) to the model that is exactly equal to *x*; *x *can then be viewed as an indicator of this latent variable. Time is incorporated into the model by populating the Λ matrix from Equation 1 with the fixed times of measurements (*t*_*j*_).

It can be shown that for a given outcome, *Y*_*j*_, at time *t*_*j*_, the predictive formula is:(5)

The interpretation of the key model parameters of interest are as follows:

1. *b*_13 _is the effect of the mediating variable on the latent intercept.

2. *b*_23 _is the effect of the mediating variable on the latent slope.

3. *γ*_33 _is the effect of the main independent variable on the mediating variable.

4. *γ*_23 _is the effect of the main independent variable on the latent slope.

5. *γ*_13 _is the effect of the main independent variable on the latent intercept.

Under the assumption of MVN errors in both the measurement and structural model, the distribution of **Y **is MVN as well. The mean for any given *Y*_*j *_is:

and the covariance matrix for the vector of *Y*_*j*_'s is:

where

The primary setting we considered in our simulations assumed a constant effect of heavy alcohol use (the main independent variable of interest) over time. If there is no alcohol by time interaction (i.e. *γ*_23 _= 0 and *b*_23 _= 0), the total effect of heavy alcohol use on CD4 count is represented by *γ *_13 _+ *b*_13 _*γ*_33_, which is the sum of its direct (*γ*_13_) and indirect (*b*_13 _γ_33_) effect through ART adherence.

Secondary analyses assuming the effect of heavy alcohol use changes over time were also performed. In this setting, the interaction between alcohol and time is the primary interest. The total effect of alcohol use on the change of CD4 count over time is represented by coefficients corresponding to the interaction between alcohol use and time, *γ*_23 _+ *b*_23 _*γ*_33_, the sum of the direct and indirect effects, respectively.

### Linear Mixed Effects Models

The LMM can be used to evaluate the total effect of heavy alcohol consumption on CD4 cell count, however, the mediated relationship is not explicitly modeled with a single LMM. Two mixed models were considered for comparison to the SEM. The first model, which we will refer to as LMM1, includes the mediator (*x*) as a covariate in the model. In the formula below we have again eliminated the subject index (*i*) for simplicity and let *j *= 1,..., 6 index time-point. The first model is:(6)

where *β*_*j*_'s are unknown regression coefficients relating covariates to the mean of *Y*_*j*_, *r*_1 _and *r*_2 _denote the random intercept and random slope, respectively and *e*_*j *_is the random error with zero mean representing deviation of responses from the corresponding predicted means. In matrix form **e **= (*e*_1_, *e*_2_,..., *e*_6_)^*T *^is the vector of unknown random errors with E(**e**) = **0 **and Cov(**e**) = **E**; **r **= (*r*_1_, *r*_2_)^*T *^is the vector of the random intercept and slope coefficients with E(**r**) = **0 **and Cov(**r**) = **G**. In the primary setting we explore, where the effect of alcohol is assumed constant and therefore the alcohol by time interaction in (6) is excluded, the effect of the main independent variable on the outcome is represented by *β*_2_. In the secondary setting explored where there is an alcohol by time interaction, the parameter of interest is *β*_5_. Variables in the causal pathway are often omitted as they otherwise artificially attenuate the effect of the main independent variable of interest. Thus, we also considered a second mixed model, which we refer to as LMM2, where we refit the model given in Equation 6 excluding the term for ART adherence (*β*_4_*x*).

The LMM assumes MVN errors, therefore the distribution of **Y **is MVN as well. For the LMM1 in Equation (6), the mean for any given *Y*_*j *_is:

and the covariance matrix of the vector of *Y*_*j*_'s is:

where

In a comparison of the means of the SEM and LMM under the assumption of multivariate normality, there are two notable differences. First, the mean for the SEM is explicitly modeled as a function of both the direct and indirect effects of the main independent variable *z*_2 _(i.e. *γ*_13 _+ *b*_13 _*γ*_33_) whereas in the LMM it is simply a function of its total effect (i.e. *β*_2_). Second, in a LMM that includes the mediating variable (LMM1), the mean of the outcome is a function of the mediating variable itself, but this is not the case for the SEM where the mean depends instead on the effect of the mediating variable on the latent intercept and slope (i.e., *b*_13 _and *b*_23_). With regard to covariance matrices, in the LMM the covariance depends on the values of time and the covariance matrix of the latent intercept and slope. In contrast, the covariance from the SEM depends explicitly on the mediating variable and its effects. That is, it is a function of the values of time, the parameters associated with the effect of the mediating variable on the random intercept and random slope, as well as the covariance matrix of the latent intercept, slope, and the mediating variable. Thus, the magnitude of the mediated effect and the covariance of the mediator and latent variables influence the covariance of *Y *in the SEM but this is not the case in the LMMs.

We simulated data under the mediated SEM and then fit the data with both the LMM1 and LMM2 models. The SEM model was also fit as a reference standard to compare with the LMM results. The goal was to identify advantages and disadvantages of using the LMM relative to the SEM in a longitudinal data setting where a mediator was present. In evaluating model performance, we focused on the parameters representing the total effect of heavy alcohol use on CD4 cell count.

### Data Characteristics to be Varied in Simulated Data

We simulated datasets assuming the mediated longitudinal relationship described in the path diagram (Figure 2). The data were generated under the SEM model as the objective was to evaluate the performance of the mixed model when the SEM is expected to be preferable. The factors we evaluated were sample size, effect size and distributional assumptions.

#### Sample Size

A range of sample sizes was evaluated. We considered sample sizes as small as 25 and increased values up to 500 at which point both the SEM and LMM performed well.

#### Effect Size and Total Effect Distribution

When the effect of heavy alcohol use is constant over time, the total effect of the main independent variable on the latent intercept is given by *γ*_13 _+ *b*_13 _*γ*_33_. We defined the effect size by scaling this quantity by the total standard deviation of the latent intercept:(7)

where

When the effect of heavy alcohol use changes over time, the direct effect of the main independent variable on the latent intercept is set to zero, and thus the effect size on the latent slope was defined as:(8)

where

We examined a range of effect sizes including small (approximately 0.2), medium (approximately 0.5) and large (approximately 0.8), as defined by Cohen [19]. In addition, within each effect size we varied the distribution of the direct and indirect effects and explored the following three scenarios: equally distributed direct and indirect effects; primarily direct effect; primarily indirect effect.

#### Distribution of the Outcome Variable

Both SEMs and LMMs assume normally distributed errors of the outcome variables. We compared the performance of each type of model when this assumption was not met. For each distribution evaluated we considered two scenarios: i) only the errors from the measurement model were non-normal and ii) errors of both the measurement and structural models were non-normal. The following distributions were evaluated:

1. Uniform() distribution- the parameters of the uniform distribution were chosen to obtain a mean of zero and a variance of one to be comparable to the standard normal setting.

2. Lognormal(0, ) distribution- the log-normal parameters were chosen such that the mean of the residual errors was equal to zero and the variance was equal to one. To achieve a mean of zero, exp(0.4812/2) was subtracted from all generated lognormal values.

3. Contaminated normal(0.4, 10) distribution- a mixture of a standard normal and normal with variance of 10, where 40% of the data were from the latter distribution [20].

4. Fleishman/Mattson method. The Fleishman [21] method describes a way to generate non-normal random variates with known skewness and kurtosis. The Mattson [22] method provides a way to generate non-normal random variates with specified correlation from non-normal random variates with known skewness and kurtosis. The method also provides a formula for the skewness and kurtosis of the randomly generated correlated values. The combined method [23] allowed us to change only the distribution of the errors while keeping the overall variance and correlation the same. We used two Fleishman/Mattson distributions. The first had a moderate level of skewness and low level of kurtosis. The second was highly skewed and highly kurtotic. The first results in a variance of 1, a skewness of 0.75 and kurtosis of 0 for the residual errors of the measurement model and skewness of (0.53, 0.5 and 0.75) and kurtosis of (-1.5, -1.9 and -3) for the three residual errors of the structural model, respectively. The second Fleishman/Mattson distribution we used results in a variance of 1, skewness of 1.75 and kurtosis of 3.75 of the residual errors of the measurement model and skewness of (1.2, 1.4 and 1.8) and kurtosis of (0.4, 0.5 and 0.8) for the three residual errors of the structural model, respectively.

### Data Simulation

To generate a dataset under the mediated SEM data structure, the following steps were taken.

1. Two multivariate normal random variates were generated, one to be the residual error of the latent intercept and one to be the residual error of the latent slope. When evaluating the impact of distributional assumptions, the non-normal distributions defined in the previous section replaced the multivariate normal distribution in this step. The Mattson method [22] was used to keep the covariance between the random intercept and random slope at the same level as was used for the normal simulations.

2. The value of the latent intercept and latent slope were computed according to the structural model given in Equation 4.

3. Independent normal errors were created to be the residual errors for each of the repeated measures of outcome. When evaluating the impact of distributional assumptions, the non-normal distributions defined in the previous section replaced the multivariate normal distribution in this step.

4. The values of the longitudinal outcome variables were computed according to the measurement model given in Equation 3.

5. Steps 1 through 4 were repeated 1000 times to create 1000 datasets.

6. Each generated dataset was fit with the SEM, the LMM1 (i.e. with the mediator as a covariate), and the LMM2 (i.e. without the mediator as a covariate).

7. Model performance was assessed with the following: i.) Bias- the difference between the true parameter value and the mean observed parameter value divided by the true parameter value. ii.) Coverage probability - the percentage of the 1000 95% confidence intervals that contained the true parameter value. iii.) Power - the percentage of the 1000 datasets in which a hypothesis test of the parameter of interest was statistically significant. With a sample size of 1000, and a true power of 80%, the width of a 95% confidence interval around a power estimate based on the simulations would be approximately 5.0 percentage points. For a true 95% coverage probability, the width of a 95% confidence interval around a coverage probability estimate would be approximately 2.7 percentage points.

## Results

The results from the mixed effects models focus primarily on the models that do not adjust for the mediator (i.e., LMM2) because these models capture the total effect of the main independent variable. Results from the mixed model adjusting for the mediator (LMM1) appear to capture the direct rather than total effect of the primary independent variable and are therefore only included in the sample size results to demonstrate this result. However, since the primary objective of the analysis was to evaluate the total effect of the main independent variable, we present only the comparison of the SEM and the mixed model that excludes the mediator in the remainder of the results. Typically, variables associated with the outcome are included in a model, including independent predictors and confounders. However, because mediators are in the causal pathway, it is recommended that such variables be excluded from a model to avoid attenuating the true association between an exposure and outcome [24]. Thus the LMM2 model is consistent with the general practice of excluding as a covariate variables thought to be in the causal pathway.

### Sample Size

The results from the sample size variation are displayed in Table 1. With sample sizes of 25, 50 and 100, the estimated power to detect the total effect for all models was quite low (14%-65%). We note that for the SEM, with a sample size of 100, the power for the total effect was 65%, while the power to detect the direct and indirect effects were 26% and 71% (data not shown), respectively. With a sample size of 200, the power for the SEM and LMM2 were both high and similar in magnitude (91% and 89%, respectively), although the power for the LMM1 remained low at 41%. The estimated power of the LMM1 for a sample size of 500 was less than 80%. With a sample size of 500 the power for the SEM and LMM2 models were 99%, therefore we did not evaluate larger sample sizes.

**Table 1:** Impact of sample size.

Simulated Data	Mediated SEM			LMM with Mediator as Covariate LMM1			LMM without Mediator LMM2		
**Sample Size**	**Bias (%)**	**Coverage Probability (%)**	**Power (%)**	**Bias (%)**	**Coverage Probability (%)**	**Power (%)**	**Bias (%)**	**Coverage Probability (%)**	**Power (%)**

25	1.5	92	26	-48	85	14	2.1	91	26

50	1.5	93	41	-49	84	16	2.0	93	42

100	1.2	94	65	-49	75	26	1.7	94	65

200	-1.2	96	91	-51	56	41	-1.1	95	89

250	-1.6	94	95	-52	48	47	-1.6	93	94

500	-1.0	95	100	-51	21	78	-1.0	95	100

Impact of sample size on model performance in evaluating the total effect of the main independent variable on random intercept.

Based on 1000 simulated datasets with medium effect size equally distributed between direct and indirect effects.

The estimated coverage probabilities for the total effect for all sample sizes for the SEM were high, ranging from 92% to 96%. For the LMM2, these coverage probabilities range from 91% to 95%. For the LMM1, the coverage probabilities were much lower than either the SEM or the LMM2 and decreased with increasing sample size. This trend is likely due to the large bias of the estimate for the LMM1 and resulting confidence intervals that are not centered at the true value. Thus the wider confidence intervals from smaller sample sizes are more likely to include the true value.

Because the model performance was good for the SEM and LMM2 with a sample size of 250 subsequent simulations evaluating effect sizes and distributional assumptions were conducted using this sample size.

### Effect Size and Total Effect Distribution

The results from the set of simulation studies varying the effect of the main independent variable on the random intercept are displayed in Table 2.

**Table 2:** Impact of effect size and effect distribution.

Simulated Data			Mediated SEM			Mixed Model without Mediator		
**Effect Size**	**Effect Distribution**	**Effect**	**Bias (%)**	**Coverage Probability (%)**	**Power (%)**	**Bias (%)**	**Coverage Probability (%)**	**Power (%)**

Large	Equal	Total	-0.8	94	100	-0.8	94	100
		
	Direct	Total	-0.8	94	100	-0.8	93	100
		
	Indirect	Total	0.9	94	100	-0.6	94	100

Medium	Equal	Total	-1.5	94	95	-1.5	93	94
		
	Direct	Total	-1.5	94	97	-1.5	93	96
		
	Indirect	Total	-1.3	94	93	-1.3	93	92

Medium-Small	Equal	Total	-2.0	94	80	-2.0	93	80
		
	Direct	Total	-0.9	94	84	-0.9	93	83
		
	Indirect	Total	-1.8	94	80	-1.8	93	79

Small	Equal	Total	-3.7	94	32	-3.7	93	32
		
	Direct	Total	-3.9	94	36	-4.0	93	35
		
	Indirect	Total	-3.7	94	34	-3.7	93	33

The impact of effect size and its distribution on model performance in evaluating the total effect of the main independent variable on the random intercept.

Based on 1000 simulated datasets with a sample size of 250.

In evaluating the effect of the main independent variable on the random intercept, the power for the SEM and the LMM2 (i.e. LMM without the mediating variable) was > 99% when the effect size was large, regardless of whether the effect was primarily direct, primarily indirect or equally distributed between the direct and indirect paths. The coverage probabilities were also very similar between the SEM and LMM2 (≥ 93% in all cases).

For a medium effect, the point estimate of power was slightly higher for the SEM compared to the LMM2 regardless of how the effect was distributed. For example, when the effect was equally distributed the power was 94% for the LMM2 and 95% for the SEM. Although the power for all models was high (≥ 92%), the power for the SEM and the LMM2 appeared to increase as the proportion of the direct effect increased. When the effect was primarily indirect the power was 92% for the LMM2 and 93% for the SEM. The coverage probabilities for the total effect were again ≥ 93% for both the SEM and the LMM2. The higher point estimates of power in the SEM appeared to be due to the larger standard error of the effect estimate in the LMM2. Similar trends were observed with the medium-small and small effect sizes although the power for all models dropped markedly with the small effect size. For example, power was approximately 32% for both models in the case of a small effect size, equally distributed between direct and indirect effects.

### Distributions

In simulations evaluating the effect of distributional assumptions, we used a sample size of 250 and a medium-small effect size that was equally distributed in direct and indirect effects (see Table 3). Results from the model with a normal distribution (and the same sample size and effect distribution as described above) had power of 80% and bias of -2.0% for both the SEM and the LMM2 and a coverage probability of 94% for the SEM and 93% for the LMM2. This is referred to below as the normal comparison model.

**Table 3:** Impact of distributional assumptions.

Simulated Data			Mediated SEM			Mixed Model without Mediator		
**Distribution**		**Non-normal Residual Error**	**Bias (%)**	**Coverage Probability (%)**	**Power (%)**	**Bias (%)**	**Coverage Probability (%)**	**Power (%)**

Uniform		Measurement	0.7	96	82	-0.8	96	81
	
		Measurement & Structural	1.4	97	87	1.6	96	85

Log-normal		Measurement	-1.3	94	82	-1.3	94	80
	
		Measurement & Structural	-0.7	95	82	-0.7	95	81

Contaminated Normal		Measurement	-0.2	95	18	0.07	95	17
	
		Measurement & Structural	9.5	96	8	11.1	95	8

Fleishman/Mattson 1		Measurement & Structural	-2.9	94	79	-3.0	94	78

Fleishman/Mattson 2		Measurement & Structural	-2.4	94	80	-2.6	94	78

The impact of distributional assumptions on model performance in evaluating the total effect of the main independent variable on the random intercept.

Based on 1000 simulated datasets with a medium-small effect size, equally distributed and a sample size of 250.

The Fleishman/Mattson 1 distribution is moderately skewed and slightly kurtotic.

The Fleishman/Mattson 2 distribution is highly skewed and kurtotic.

Assuming a uniform distribution on the residual errors of the measurement model the power to detect the total effect was estimated to be 82% for the SEM and 81% for the LMM2. Both of these models had similar estimates of power which were slightly greater than the power estimate of the comparison models with normal residual errors. This was likely due to an underestimation of the standard error of the parameters. For the model with normal residual errors, the mean of the standard errors of the total effect was 0.16 whereas the mean of the standard errors for the uniform was 0.15. The coverage probability was very similar for both the SEM and the LMM2 (96%). These estimates were slightly higher than those for the normal comparison models. The bias was small (< 1%) for both SEM and LMM2. The results were similar when a uniform distribution was used for the residual errors of both the measurement and structural models.

Model performance was good overall for both the SEM and LMM2 when errors followed a log-normal distribution (see Table 3), although not as good as under the uniform distribution.

Power declined noticeably for both models when errors of the measurement model followed a contaminated normal distribution, however, the coverage probability and bias remained good (power ≤ 18%, coverage probability ≥ 95% and bias ≤ 0.2%). The coverage probability remained high likely due to the large standard error estimates yielding wide 95% confidence intervals. Similar trends were observed for the contaminated normal distribution on the residual errors of the measurement and structural models although model performance declined for both the SEM and LMM2. The lower power of both the SEM and the LMM2 fit to the contaminated normal data may be explained by the relatively large values of the residual variances created by the contaminated normal. For example, the estimated mean values for the residual variance was 40.6 in the SEM and the LMM2 compared to around 1 in the models based on a normal distribution. The effect of a large residual variances is a decrease in the true effect size of the main independent variable.

Both models performed well when measurement and structural errors followed Fleishman/Mattson distribution. The power was similar for the SEM and LMM2 (79% and 78%, respectively). Both models had the same coverage probabilities and bias, 94% and -3%, respectively. Similar values and trends were seen with the second Fleishman/Mattson distribution.

The results of an SEM are generally presented with at least two fit indices [25]. Commonly used fit statistics are the chi-square statistic, the AIC, the root mean square error of approximation (RMSEA) and the standardized root mean square residual (SRMR). LMM models are not usually presented with fit statistics, although during model specification, fit indices like the log-likelihood, AIC and BIC have been used for model selection [26]. The fit statistics for the SEM and LMM2 under different error distributions for both the measurement and structural models are given in Table 4.

**Table 4:** Goodness of fit.

		Distribution			
**Model**	**Fit Statistic**	**Uniform**	**Lognormal**	**Fleishman/Mattson 1**	**Fleishman/Mattson 2**

SEM	Chi-square	36.6	58.0	38.4	43.7
	
	RMSEA	0.010	0.040	0.023	0.021
	
	SRMR	0.039	0.043	0.041	0.041
	
	AIC	7309	7288	7307	7307

Mixed Model without Mediator	-2LogLikelihood	5550	5537	5549	5547
	
	AIC	5566	5553	5565	5563
	
	BIC	5594	5581	5593	5591

Assessing goodness of fit of SEMs and LMMs with datasets with non-normal error distributions.

Normal comparison model has SEM values of Chi-square = 38.2, RMSEA = 0.012, SRMR = 0.040, AIC = 7306 and LMM values of Negative 2Loglikelihood = 5548, AIC = 5564 and BIC = 5592.

Lower values suggest better model fit for each of the fit statistics presented. RMSEA values of < 0.05 and SRMR values of < 0.1 are considered good fit [25]. However, all of the fit statistics from the SEM with the exception of the AIC indicate that the log-normal, the most skewed distribution, had the worst fit followed by the second Fleishman/Mattson model which also has a skewed distribution. The AIC from the SEM and all of the fit statistics from the LMM2 were less affected by the skewness of the log-normal.

### Results when Effects of the Main Independent Variable Change Over Time

The simulation results for the effect of the main independent variable on the random slope did not differ qualitatively from the results for the random intercept (data not shown). Overall, the point estimates of power were slightly higher for all models (SEM and LMM) likely due to the fact that no covariate by time interaction was included in the models. The lack of this additional interaction term results in higher true power for the effect of the main independent variable on the random slope. In general, the simulation results were similar to those observed in the primary setting where the main independent variable had a constant effect across time.

## Discussion

Linear mixed effects models are often used to analyze longitudinal data. Although LMMs can be applied in settings where mediation is present, it is unclear whether they perform sufficiently well relative to SEMs which have a framework that explicitly allows for mediational analyses. The objective of this paper was to evaluate the performance of the LMM in the analysis of longitudinal data with a single mediating variable, a setting conducive to the use of SEMs.

The simulation studies were conducted to assess whether the mixed effects model adequately modeled the mediated longitudinal relationships or if employing SEMs was necessary. The LMM and SEM were compared under a range of settings evaluating sample size, effect size and distributional assumptions. The results of our simulation study suggest that the mixed effects model performs comparably to the SEM with respect to power, bias and coverage probability in the analysis when the objective is to estimate the total effect of a primary independent variable. In addition, we demonstrated that mixed effects models used for the purpose of estimating total effects should not include mediating variables as covariates, since resulting coefficients represent the direct effect of the main independent variable on the outcome and erroneous conclusions could be drawn if these effects were interpreted as the total effect.

Both the LMM and SEM were robust to violations of the normality assumption. For the SEM, lack of normality had a larger impact on the model fit statistics than on power, coverage probability and bias. The uniform distribution, an example of a kurtotic, but not skewed distributions, had little effect on the SEM fit statistics compared to the negative impact observed for the lognormal and Fleishman/Mattson distributions, which were both skewed and kurtotic. Generally, the highest levels of skewness had the worst fit. For this reason, caution should be used in applying the SEM when the normality of the data is in question, particularly if the distribution of the data is skewed.

There are several considerations in deciding whether to use an SEM or a mixed model to analyze longitudinal data when a mediating factor is present. The SEM may provide a marginal increase in power, although the difference may not be statistically significant. More importantly, it efficiently evaluates the mechanism of the total effect, decomposing direct and indirect pathways, in a single model. However, larger sample sizes are required to make inferences about the specific direct and indirect effects. If the sample size is limited and the goal is to evaluate only the total effect of a primary independent variable, rather than delineating direct versus indirect effects, then the mixed model provides similar power and coverage probability to the SEM. Although direct and indirect effects could be evaluated by fitting additional mixed models (e.g. models with and without mediating factors), it is a less efficient approach compared to the SEM. In addition, there are broader issues that may influence choice of model such as clinical context, study design and sample size.

Complex SEMs may be difficult to implement without specialized software. Although common software packages such as SAS and R have the capability to run SEMs, software designed specifically for SEMs (e.g. Mplus, LISREL and AMOS) may be more intuitive and user-friendly in model specification, particularly in the development of highly complex models.

The current study examines one specific setting of mediated longitudinal data. Other situations with different data structures where mediation is present could also be explored, e.g. situations where the mediator and the primary independent variable as well as the outcome are repeatedly measured, categorical outcomes, and settings with more complex pathways between variables. In addition, we specifically explored the question of whether the LMM performs sufficiently in a setting favorable to the SEM. Future studies examining broader settings where the data arise from non-SEMs would provide further insight into the use of the LMM and SEM in mediated longitudinal settings.

## Conclusions

In general, both SEMs and LMMs were robust methods with similar power in a variety of scenarios. The main advantage of the SEM is the ability to estimate the direct and indirect pathways of the effect of the primary independent variable on the outcome, given sufficient sample sizes. Despite not directly modeling the mediated pathways, LMMs excluding mediating variables performed well with respect to power, bias and coverage probability in modeling the total effect of the primary independent variable on the outcome.

## Competing interests

The authors declare that they have no competing interests.

## Authors' contributions

All authors read and approved the final manuscript. EAB was involved in the conception of the study, designing and performing the simulation analysis and drafting the manuscript. HC and TH were involved in conception of the study, designing the simulation study and critically revising the manuscript. DMC was involved in conception of the study, designing the simulation analysis and drafting the final manuscript.

## Pre-publication history

The pre-publication history for this paper can be accessed here:

http://www.biomedcentral.com/1471-2288/10/16/prepub
